# A Comprehensive Policy Landscape for Alzheimer’s and Related Dementias: Past, Present, and Future Directions

**DOI:** 10.1093/geroni/igaf122.1724

**Published:** 2025-12-31

**Authors:** Lauren Stratton, Robert Egge, Rachel Conant, Jennifer Rosen, Matthew Baumgart, Carl Hill, Lycia Neumann, Katie Evans

**Affiliations:** Alzheimer’s Association, Chicago, Illinois, United States; Alzheimer’s Association, Chicago, Illinois, United States; Alzheimer’s Association, Chicago, Illinois, United States; Alzheimer’s Association, Chicago, Illinois, United States; Alzheimer’s Association, Chicago, Illinois, United States; Alzheimer’s Association, Chicago, Illinois, United States; Alzheimer’s Association, Chicago, Illinois, United States; Alzheimer’s Association, Chicago, Indiana, United States

## Abstract

The Alzheimer’s Association, as the leading voluntary health organization in Alzheimer’s care, support, and research, presents a detailed analysis of the evolving policy landscape surrounding Alzheimer’s and related dementias (ADRD) in the United States. Federal and state level policies have been pivotal in advancing ADRD research. The passage of the National Alzheimer’s Project Act (NAPA) marked a central moment, establishing the first National Plan to Address Alzheimer’s Disease and creating the Advisory Council on Alzheimer’s Research, Care, and Services, and has since led to subsequent policy achievements. Additionally, this framework has been furthered by three key reauthorization acts: the NAPA Reauthorization Act, the Alzheimer’s Accountability and Investment Act, and the BOLD Infrastructure for Alzheimer’s Reauthorization Act. These legislative successes have resulted in unprecedented increases in federal research funding and substantial improvements in public health infrastructure. This presentation will explore the current policy implications and future directions through multiple lenses, including research and science, public health initiatives, and care and support advancements. Emphasizing the importance of comprehensive care models and equitable access to resources, strategic initiatives to sustain momentum in ADRD research, risk reduction, diagnosis, and access to treatment will be discussed. These initiatives include increased funding, nationwide awareness campaigns, and standardized care protocols. The session will conclude with a discussion on potential federal and state legislative actions and funding priorities to support ongoing and future efforts in combating ADRD.

